# Alterations in the gut microbiome with hemorrhagic transformation in experimental stroke

**DOI:** 10.1111/cns.13736

**Published:** 2021-09-30

**Authors:** Qin Huang, Liao Di, Fang Yu, Xianjing Feng, Zeyu Liu, Minping Wei, Yunfang Luo, Jian Xia

**Affiliations:** ^1^ Department of neurology Xiangya Hospital Central South University Changsha Hunan P.R. China; ^2^ Hunan Clinical Research Center for Cerebrovascular Disease Changsha China

**Keywords:** gut microbiota, gut‐brain axis, hemorrhagic transformation, hyperglycemia, stroke

## Abstract

**Objective:**

Hemorrhagic transformation (HT) is a life‐threatening complication of stroke. Whether changes in gut microbial composition underlie the development of HT remains unknown. This study aimed to investigate whether the gut microbiota is altered in HT rats and examine the association between these changes and inflammatory responses.

**Methods:**

HT was successfully established in rats injected with 50% glucose (6 ml/Kg, i.p.) 15 min before middle cerebral artery occlusion (MCAO, 90 min occlusion) with reperfusion. After 5 days, rats were euthanized, and their brains used to estimate infarct volume. The inflammatory factors, the analysis of gut microbiota, and short‐chain fatty acids (SCFA) were assessed.

**Results:**

In contrast with non‐HT rats, gut microbiota sequencing showed an elevation in the relative abundance of Proteobacteria and Actinobacteria in HT rats. Total SCFAs, especially butyrate and valeric acid, were significantly lower in the cecal contents of HT rats than in those of non‐HT rats. Hyperglycemia‐induced HT exacerbation was not observed when rats were treated with antibiotics, suggesting that altered microbiota play a critical role in hyperglycemic HT pathogenesis. Furthermore, rats whose gut was colonized with HT rat microbiota showed increased susceptibility to HT.

**Conclusion:**

This study provides important information about the gut microbiota profiles and SCFA levels of MCAO rats with HT or non‐HT. The susceptibility to HT in MCAO rats is associated with inflammation and gut microbiota modulation.

## Graphical abstract

The gut microbiota modulates the inflammatory response and MMP9 that are associated with hemorrhagic transformation after stroke. Hemorrhagic transformation (HT) leads to gut dysbiosis and the inflammatory response. The changes of gut microbiota, peripheral inflammation, and SCFAs may further regulate MMP9 level and affect blood‐brain barrier permeability, contributing to an increase in HT susceptibility after stroke. MCAO rats that received fecal microbiota transplantation (FMT) of the “HT microbiota” exhibited high risk of HT.

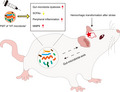

## 1 INTRODUCTION

Hemorrhagic transformation (HT) is a problematic complication of cerebral ischemic infarction (1). Hyperglycemia within 24 h of the onset of cerebral ischemia has been linked to the exacerbation of cerebral damage and increased morbidity. In addition, hyperglycemia can dramatically aggravate HT in the rat middle cerebral artery occlusion (MCAO) model (2). Possible involved mechanisms may be inflammation and oxidative stress, which further contribute to blood‐brain barrier (BBB) disruption (3). Matrix metalloproteinases (MMPs), like MMP‐2 and MMP‐9, which are zinc‐binding proteolytic enzymes that remodel the extracellular matrix, are related to BBB disruption, edema formation, and HT (4). Therefore, maintaining BBB integrity and preventing inflammation can reduce the incidence of HT (5).

Research on gut microbiota has recently increased due to technological advances in bioinformatics and DNA sequencing. Several studies have revealed bidirectional communication in stroke patients. Stroke alters gut microbiota composition, and in turn, gut dysbiosis has an impact on stroke outcomes (6). Recently, a prospective case‐control study revealed higher disruption of intestinal communities, followed by a loss of butyrate‐producing bacteria, during ischemic and hemorrhagic stroke compared with non‐stroke matched control subjects (7). Sodium butyrate, a short‐chain fatty acid (SCFA), has been shown to have anti‐inflammatory and neuroprotective effects in stroke (8), while glucose promotes the abundance of mucolytic bacteria, facilitates degradation of the mucus barrier, and triggers inflammatory responses (9). However, although gut microbiota has been associated with the development of stroke, their relationship with HT after stroke remains unclear. Therefore, the present study aimed to investigate the interaction between gut microbiota and HT after stroke.

## 2 MATERIAL AND METHODS

### 2.1 Animals and HT induction

The Institutional Animal Care and Use Committee of the central south university approved all experimental protocols (approval No. 2019–0004) on July 8, 2020. All experimental procedures were in accordance with the laboratory animal welfare and use set by the Ministry of Health of China and the National Institutes of Health (NIH) guidelines. All animal data reporting has followed the ARRIVE guidelines (10). Forty‐four male Sprague‐Dawley (SD) rats (300 to 320 g, 7–8 weeks old) were purchased from Hunan SJA Laboratory Animal Co. Ltd (Changsha, China). All the animals were generated in specific pathogen‐free (SPF) levels and housed in standard conditions (daily cycles of 12 h of light at 23℃ and 12 h of dark at 21℃, 50%–55% humidity, light intensity of 100–200 L ux, and ad libitum access to sterilized tap water and commercial rodent food). After acclimatization for 1 week, rats were randomly divided into the following groups: control, sham, hyperglycemic (HG) group, and normoglycemic (NG) group. Rats in the HG group were injected intraperitoneally with 50% dextrose (6 mL/kg) at the beginning of the MCAO procedure to induce acute hyperglycemia (1). Rats in the NG group received 0.9% NaCl via the intraperitoneal (IP) route. Sham‐operated rats were injected IP with 50% dextrose and underwent the same surgical procedures but without suture insertion. Rats without any operation served as controls. The hyperglycemic state was confirmed by detecting blood glucose levels, which showed severe hyperglycemia (250– 300 mg/dL) at 15 min after injection. This level was significantly higher than in the normoglycemic state (100–180 mg/dL). To measure blood glucose levels, tail venous blood was sampled: (1) before surgery, (2) immediately after MCAO procedure, (3) 45 min after MCAO, (4) at reperfusion, and (5) 1 h after reperfusion.

### 2.2 Middle cerebral artery occlusion model

Middle cerebral artery occlusion followed by reperfusion was performed as previously reported (11). Briefly, the rats had a supine position, and the pterygopalatine artery, internal carotid artery (ICA), and the external carotid artery (ECA) were exposed. The right external carotid artery was isolated and ligated. A 4–0 silicone‐coated monofilament nylon suture with a round tip was inserted into the ICA through the external carotid stump and advanced to occlude the origin of middle cerebral artery. Reperfusion was produced by gently withdrawing the suture out of the ECA. That is, the suture was removed at 90 min after occlusion, and the neck incision was closed. All the animals were housed individually and killed after 5 days for further analysis.

### 2.3 Neurological evaluation

The rats were neurologically evaluated in a blinded fashion about neurological deficit caused by the brain ischemia, just as reported by Garcia et al (12). Briefly, neurological evaluations were carried out once a day before the end of each experiment. When the rats recovered from anesthesia, they were also evaluated on neurological deficit. The neurological evaluation includes six tests such as spontaneous activity (in cage for 5 min), symmetry of movements (four limbs), symmetry of forelimbs (outstretching while held by tail), climbing wall of wire cage, reaction to touch on either side of trunk, and response to vibrissae tough. The maximum neurological score was 18, and the minimum was three. The lower the score, the more serious the neurological deficit. Neurological score was assessed by an observer who was blinded to the group assignments.

### 2.4 2,3,5‐triphenyltetrazolium chloride (TTC) staining

Rats were evaluated by decapitation, and brains were quickly removed and chilled in ice‐cold phosphate‐ buffered saline (PBS) for 5 min. Five consecutive 2‐mm‐thick coronary slices were sectioned from an 10‐mm‐thick region. TTC staining was performed to determine infarct volume at 5 d after MCAO as previously reported (13). HT was defined as the presence of visible bleeding in coronal brain sections (14). We first took pictures of the whole and sliced brain to see whether HT was present. Then, the brain slices were immersed in 2% TTC (Sigma) at 37℃ for 30 min in the dark. The infarction (unstained) area and hemisphere area of each section was traced and measured by using an Image J analysis system, version 1.32 (National Institutes of Health). The infarct area data of all sections multiplied by the thickness of sections were pooled for calculating the total infarct volume. The infarct volume was expressed as a percentage of the whole contralateral hemisphere (15). Furthermore, dead animals were counted for mortality rates.

### 2.5 Evaluation of hemorrhagic transformation and hemoglobin content

In brain slices (5 stained brain sections), macroscopic HT was scored by a blinded investigator using a four‐point rubric, as follows: 0 = no hemorrhage; 1 = HI Ⅰ, dispersed individual petechiae; 2 = HI Ⅱ, confluent petechiae; 3 = PH Ⅰ, small diffuse hemorrhage or hematoma (<30% of the infarcted area); 4 = PH Ⅱ, large diffuse hemorrhage or hematoma (>30% of the infarcted volume), total scores for each animal were calculated (1). The stained brain sections are then separated into contralateral and ipsilateral hemispheres, snap frozen, kept at −80℃ for the assays of hemoglobin content (16). Following ipsilateral hemispheres homogenized with PBS and centrifuged for 30 min (13000 g), the supernatant (40 μL) was collected and was mixed with 160 μL Drabkin's reagent (Sigma Diagnostics), and hemoglobin content for detecting brain hemorrhage was quantified by spectrophotometric assay (optical density at 540 nm). A reference curve was generated using homologous blood as previously described (17).

### 2.6 Biochemical determination

Plasma samples were obtained for MMP‐9, TNFa, IL‐1β, IL‐10, and IL‐17 assay in conformance with ELISA kits. The contents of MMP‐9, TNFa, IL‐1β, IL‐10, and IL‐17 were measured using enzyme‐linked immunosorbent assay (ELISA) kits (USCN Life Science, Wuhan, China). The absorbance at 450 nm was quantified using an Epoch Microplate Spectrophotometer (BioTek).

### 2.7 Sequencing of 16S rRNA genes

The feces of rats were collected from the cecum after euthanasia. That is, after the rats were deeply anesthetized, the feces in cecum were squeezed out and immediately frozen in liquid nitrogen and stored at −80℃ until further processing for either microbial composition or SCFA. Freshly voided stool samples were collected and frozen. Total genomic DNA was extracted using the cetyltrimethyl ammonium bromide method. DNA concentration and purity were monitored on a 1% agarose gel. According to the concentration, the DNA was diluted to 1ng/μl using sterile water. The collected cecal contents were used to analyze the microbial diversity (18). Microbial composition was assessed using Illumina's established 16S rRNA amplicon sequencing method and the MiSeq sequencing platform (19). The primer is as follows: 16S V3‐V4: 341F‐806R, ITS1: ITS1F‐ITS2R. 16S rRNA genes were amplified using a specific barcoded primer. All PCR reactions were carried out in 30 μL reactions with 15 μL of Phusion®High‐Fidelity PCR Master Mix (New England Biolabs), 0.2 μM of forward and reverse primers, and about 10 ng template DNA. Thermal cycling consisted of initial denaturation at 98℃ for 1 min, followed by 30 cycles of denaturation at 98℃ for 10 s, annealing at 50℃ for 30 s, and elongation at 72℃ for 60 s, and finally, 72℃ for 5 min. Sequencing libraries were generated using NEB Next®Ultra™DNA Library Prep Kit for Illumina (NEB, USA) following manufacturer's recommendations, and index codes added. Library quality was assessed on a Qubit@ 2.0 Fluorometer (Thermo Scientific) and an Agilent Bioanalyzer 2100 system. Finally, the library was sequenced on an Illumina Miseq/HiSeq2500 platform, and 250/300 bp paired‐end reads were generated. QIIME was used to de‐multiplex barcoded reads and perform chimera filtering. Filtered sequence reads were grouped into operational taxonomic units (OTUs) at a 97% sequence similarity level similar to species‐level phenotypes (20). OTUs taxonomy waereassigned, and sequences aligned with the RDP classifier and Pynas (21).

### 2.8 Microbiota data analysis

Statistical analysis of 16 s rDNA sequencing data was performed on alpha (reflecting intra‐ individual bacterial diversity) and beta (inter‐individual dissimilarity) diversity measurements. UPARSE software package with the UPARSE‐OTU and UPARSE‐OTUref algorithms was used to perform the sequence analysis. Sequences with ≥97% similarity were assigned into OTUs. Alpha (within samples, including three metrics: Chao1, Observed Species, and the Shannon index) and beta (among samples) diversity were analyzed using an in‐house developed Perl scripts. We pick an representative sequence for each OTU and use the RDP classifier to annotate taxonomic information for each representative sequence. In order to compute Alpha Diversity, we rarify the OTU table and calculate three metrics: Chao1 estimates the species abundance; Observed Species estimates the amount of unique OTUs found in each sample, and Shannon index. Rarefaction curves were generated based on these three metrics.

Cluster analysis was preceded by principal component analysis (PCA), which was applied to reduce the dimension of the original variables using the QIIME software package. QIIME calculates both unweighted and weighted unifrac distance, which belongs to phylogenetic measures of beta diversity. We used unweighted unifrac distance for Unweighted Pair Group Method with Arithmetic mean (UPGMA) Clustering and Principal Coordinate Analysis (PCoA). PCoA is used to get principal coordinates and visualize them from complex and multidimensional data. It takes a transformation from a distance matrix into a new set of orthogonal axes, by which the maximum variation factor is demonstrated by first principal coordinate, and the second maximum one by the second principal coordinate, and so on. UPGMA Clustering is known as a type of hierarchical clustering method using average linkage and can be used to interpret the distance matrix.

To confirm differences in the abundances of individual taxonomy between the two groups, STAMP software was utilized. Linear discriminant analysis effect size (LEfSe) was used for the quantitative analysis of biomarkers within different groups. The linear discriminant analysis threshold was set to 2. This method was designed to analyze data in which the number of species is much higher than the number of samples and to provide biological class explanations to establish statistical significance, biological consistency, and effect‐size estimation of predicted biomarkers. To identify differences of microbial communities between the two groups, ANOSIM and ADONIS were performed based on the Bray‐Curtis dissimilarity distance matrices. To predict metagenome functional content from 16S rRNA gene surveys, PICRUSt bioinformatics software was accessed, and to generate the differential pathways, the KEGG (Kyoto Encyclopedia of Genes and Genomes) pathways were used.

### 2.9 SCFA measurements

SCFA concentration in the feces was determined using gas chromatography (22). Stool (30 mg) was homogenized in 900 µL of 0.1 N hydrochloric acid. Phosphoric acid (200 µL at 25%) was then added, and the sample centrifuged at 14000 × g for ten min. The supernatant was added to an internal standard solution (500 μM of 4‐methyl‐valeric acid, Sigma‐Aldrich) and 5% phosphoric acid in a glass chromatography tube, well mixed, and kept at room temperature for 30 min. The supernatant was analyzed for SCFA using agas chromatograph (Agilent 7890A/5975C, USA) and Agilent DB‐WAX column (30m × 0.25mm × 0.25um). A flame‐ionization detector with an injector temperature of 150℃ and a detector temperature of 250℃ was employed. The concentration of acetic, propionic, butyric, iso‐butyric, valeric, isovaleric, and hexanoic acids was determined using standard curves.

### 2.10 Depletion of commensal bacteria

To inhibit the growth of commensal bacteria, rats were treated with a cocktail of antibiotics (ABX) containing ampicillin (1 g/L), vancomycin (500 mg/L), neomycin (1 g/L), and metronidazole (1 g/L) (Meilun Biotechnology, Dalian, China) in drinking water for 4 weeks. Bottles with antibiotic‐containing water were inverted every day, and the antibiotic solution changed every 1–2 days, together with the cage bedding. The ABX selection is based on previous reports showing that they effectively deplete gut bacteria and may be used to model a germ‐free condition (23, 24). Four weeks after treatment, HT was induced by 50% glucose (6 ml/Kg, IP) 15 min MCAO. ABX was continued until the rats were euthanized, 5 days after surgery.

### 2.11 Microbiota transplantation

Fecal microbiota transplantation was performed as previously reported (25). After collection of feces from HT rats, fecal homogenates were centrifuged at 1000 rpm, and the supernatant collected. Fecal supernatants were orally gavaged into microbiota‐depleted rats. As described above, rats were treated with an antibiotic cocktail for 1 week before the administration of fecal extract to deplete the microbiota (26). Food was withdrawn from recipient rats 8 h before fecal transplantation. Two weeks after fecal transplantation, the recipient rats underwent MCAO surgery.

### 2.12 Statistical analysis

GraphPaD Prism (v. 9.0) software and SPSS 20.0 (IBM, USA) was used for statistical analysis. Data are shown as mean ± SD. Data normality was tested using the Kolmogorov‐Smirnov normality test and Shapiro‐Wilcoxon normality test, rejecting normality at *p* < 0.05. Statistical differences between two groups were assessed by the unpaired Student's t test or Mann‐Whitney rank sum test. Statistical significance among more than 3 groups was determined by a one‐way analysis of variance (ANOVA) followed by Bonferroni's multiple comparison tests. Nonparametric analyses were performed with the Kruskal‐Wallis H test (more than 3 groups). Next‐generation sequencing analysis of differences of relative abundances was assessed by Tukey's honest significant difference tests by R package. Statistical significance was set at *p* < 0.05. We used a Kaplan‐Meier curve analysis to evaluate the overall survival. The relationship between intestinal microbiota and HT biomarkers was analyzed by spearman correlation analysis.

## 3 RESULTS

### 3.1 HT model evaluation

**FIGURE 1:**
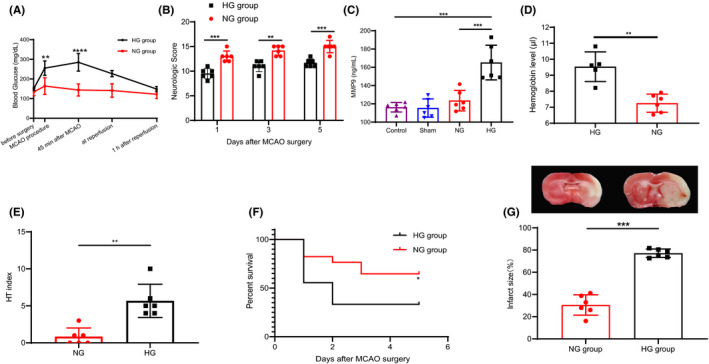
Blood glucose, HT index, neurological score, and interaction size after middle cerebral artery occlusion. (A) Level of blood glucose in the rats of different groups at different time points after dextrose administration (n = 6 rats per group). (B) Neurological scores. Grades of 3 to 18 are used. Hyperglycemia significantly decreased the neurological deficit as compared to NG group 1 day, 3 days, and 5 days after MCAO induction (n = 6 rats per group). (C) Matrix metalloproteinase 9 (MMP‐9) activity on the 5th day after MCAO surgery. Administration of 50% glucose solution achieved and maintained higher MMP‐9 levels (n = 7 to 9 per group). (D). The hemoglobin content in the ischemic hemisphere which is used as a measure of the severity of bleeding (n = 6 per group). (E) HT index, a measure of macroscopic HT occurrence, verified increased HT in hyperglycemic rats (n = 11 to 13 per group). (F) Symptom‐free curve (Kaplan‐Meier analysis curve). Hyperglycemia administration deteriorated the symptom‐free survival. (G) Acute hyperglycemia significantly increased infarct size and hemorrhage volume at days after reperfusion. The representative images of HT morphology are shown on *top*. Ratios of infarct volume, shown at the *bottom*. A significantly higher ratios of infarct volume to contralateral hemisphere volume were observed in HG group (n = 6 rats per group). Statistical analysis was performed by unpaired Student's t test in A, B, D, and G. Mann‐Whitney rank sum test was used in B and E. One‐way ANOVA analysis was used in C. Data represent means ± SD. *p < 0.05, **p < 0.01, ***p < 0.001, and ****p < 0.0001 [Colour figure can be viewed at wileyonlinelibrary.com]

To understand the effects of acute hyperglycemia on brain infarction, we injected rats with 50% glucose 15 min before performing 1.5 h MCAO with reperfusion. The blood glucose levels in all animal groups during surgery were significantly higher than at the time point of dextrose injection (Figure 1A). After 1 h of reperfusion, blood glucose dropped to control levels in all groups. The neurological score in the rats with HG group was significantly lower than that in the NG group (Figure 1B). MMP‐9, a marker of HT events in ischemic stroke, was higher in the HG group than in the NG group (Figure 1C). In the HG group, the HT index was significantly increased, demonstrating that HT induced by acute hyperglycemia was reliable (*p *= 0.002, Figure 1E). Conformably, the hemoglobin content was also higher in HG group than in NG group (Figure 1D). Hyperglycemia induced significant HT in MCAO rats and increased infarct volumes at 5 days after reperfusion when compared with normoglycemic MCAO rats (Figure 1G). Macroscopic evaluation revealed larger infarct size and dispersed individual petechiae or confluent petechiae in the HG group than in the NG group (Figure 1G). The areas under the Kaplan‐Meier curve for rat survival after 5 days in HG group and NG group were 0.25 and 0.60, respectively (Figure 1F). Together, these data suggest that hyperglycemia before MCAO predisposes to HT and exacerbates neurovascular injury.

### 3.2 HT alters gut microbiota composition

**FIGURE 2:**
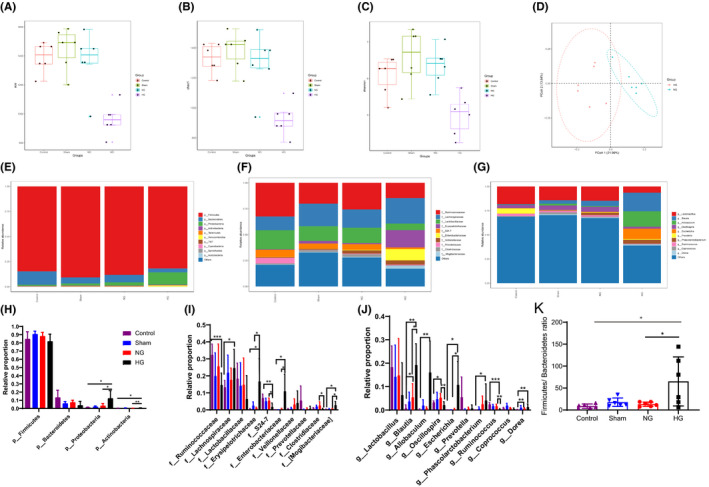
HT contribution to marked microbial dysbiosis. (A‐C) Box plot of alpha diversity of gut microbiota in rats administrated with hyperglycemia. These indexes included ace index, chao index, and shannon index (n = 6 per group). (D) Beta diversity analysis via PCoA of the HG group and NG group. PCoA reflects the differences in species bets diversity among the samples by using Bray‐Curtis distance matrix. ANOSIM: R = 0.45, *p* = 0.01 (n = 6 per group). (E to G)Taxonomic summary of the gut microbiota of different groups at (E) phylum level, (F) family level, and genus level (G). The abscissa represents the group, and the ordinate represents the relative abundance (n = 6 per group). (H to J) Comparison of the representative taxonomic abundance in different groups. One‐way ANOVA analysis or Kruskal‐Wallis H test indicated the significant differences among different groups at phylum, family, and genus level. (K) Firmicutes/Bacteroidetes ratio in different groups (n = 6). Statistical analysis was performed by one‐way ANOVA analysis or Kruskal‐Wallis H test. Date represent means ± SD. *p < 0.05, **p < 0.01, ***p < 0.001, and ****p < 0.0001 [Colour figure can be viewed at wileyonlinelibrary.com]

The above results and other studies in gut microbiota and stroke (27, 28) led to the hypothesis that hyperglycemia‐induced HT alters the composition of gut microbiota. Therefore, we characterized the microbial composition of fecal samples collected from rats for 5 days after surgery using 16S ribosomal RNA (rRNA) gene sequencing analyses. The total number of sequences used reach 5973976 reads, with an average value of 248915 reads/sample and a mean sequence length of 521 bp. Sample coverage calculation showed a satisfactory coverage for all the samples (>96%). α‐diversity using ACE, Chao, and Shannon indices showed that HT significantly reduced species richness (HG group vs. NG group, *p *= 0.003, 0.004, and 0.0015, respectively) (Figure 2A–C). The β‐diversity in both HG and NG rats was significantly altered, as reflected by distinct clustering patterns on principal coordinates analysis plots (PERMANOVA, R^2^ = 0.217, *p *= 0.008, Figure 2D).

**FIGURE 3:**
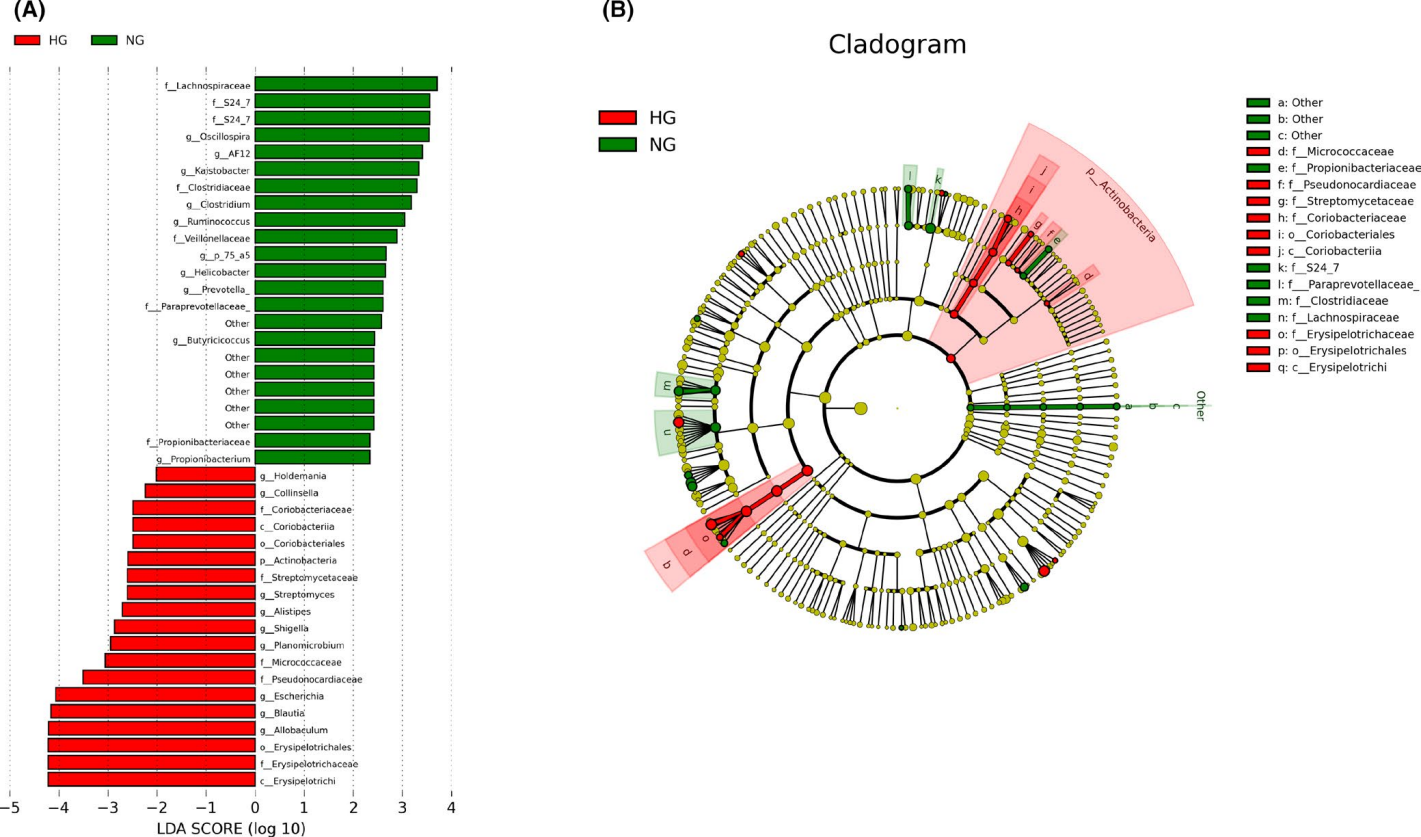
Significantly discriminative taxa among the various groups determined using linear discriminant analysis effect size (LDA effect size). (A) Taxa were sorted by degree of difference. Only the taxa meeting a significant LDA threshold value of >2.0 are shown. (B) The overall representation of bacteria composition in HG and NG groups by cladogram. All species with a relative abundance of less than 1% and classified as “unclassified” and “unidentified” were classified as “Others” (n = 6 per group) [Colour figure can be viewed at wileyonlinelibrary.com]

Microbial community barplot showed that the microbiota composition of phylum, family, and genus changed significantly (Figure 2E–G). At the phylum level, we analyzed the top ten components and found that Firmicutes (86%), Bacteroidetes (7.69%), Proteobacteria (4.7%), and Actinobacteria (0.4%) constituted the four most dominant phyla in the four groups. The *Firmicutes*/ *Bacteroidetes* (F/B) ratios were higher in NG rats than in controls (*p *= 0.034) and also higher in HG group than in NG group (*p *= 0.047; Figure 2K). Compared to the NG group, Proteobacteria (*p *= 0.047; Figure 2E, H) and Actinobacteria (*p *= 0.003; Figure 2E, H) were elevated in the HG group. At the family level, the relative abundance of Lachnospiraceae (*p *= 0.04), Erysipelotrichaceae (*p *= 0.014), Enterobacteriaceae (*p *= 0.026), and Mogibacteriaceae (*p *= 0.028) increased compared to the control group, whereas Ruminococcaceae (*p *= 0.001) and S24–7 (*p *= 0.004) decreased in the HG group. In contrast to the NG group, S24–7 (*p *= 0.025), and Clostridiaceae (*p *= 0.048) were significantly decreased in the HG group, while Erysipelotrichaceae (*p *= 0.017), Enterobacteriaceae (*p *= 0.031), and Mogibacteriaceae (*p *= 0.049) were significantly elevated (Figure 2F, I). In terms of microbial composition at genus level, hyperglycemia‐induced HT decreased the relative abundance of *Ruminococcus* (*p *= 0.0001) and increased the relative abundance of *Blautia* (*p *= 0.004), *Allobaculum* (*p* = 0.002), Escherichia (*p* = 0.027), *Phascolarctobacterium* (*p* = 0.041), and Dorea (*p* = 0.009) compared with the control rats. In contrast to the NG group, the relative abundance of *Blautia* (*p* = 0.011) was increased while the relative abundance of *Oscillospira* (*p* = 0.008), *Allobaculum* (*p* = 0.02), *Escherichia* (*p* = 0.026), and *Ruminococcus* (*p* = 0.007) was significantly decreased in the HG group (Figure 2G, J). Next, the microbiol markers between HG and NG group were identified by linear discriminant analysis effect size (LEfSe). In our study, a total of 35 differentially abundant taxons (from phylum to genus level) were found between HG and NG group (Figure 3A, B). We also identified the microbiol markers between HG and control group by LEfSe analysis (shown in supplementary Figure 1). Specifically, there are 30 increased abundant taxons in HG group compared with control rats, such as Actinobacteria, Proteobacteria, Verrucomicrobia, Synergistetes, *Erysipelotrichaceae*, *Butyricimonas*, and *Desulfovibrio*.

### 3.3 Effect of HT on SCFA cecal levels

**FIGURE 4:**
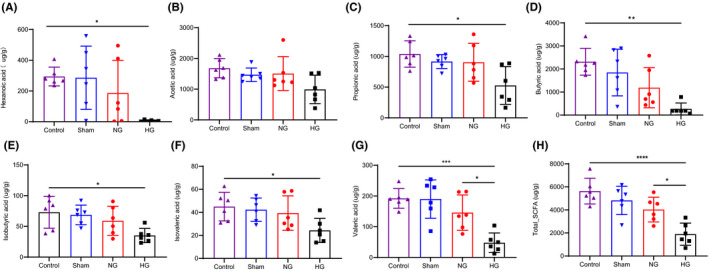
HT in experimental stroke influenced the levels of fecal SCFAs in rats. (A‐H) Fecal short‐chain fatty acids concentrations are depleted in HT rats including hexnoic acid, acetic acid, propionic acid, butyric acid, lsovaleric acid, and valeric acid. Data are shown as mean ± SD. Statistical significance was considered at *p﹤0.05, **p﹤0.01, and ***p﹤0.001 for one‐way ANOVA analysis or Kruskal‐Wallis H test [Colour figure can be viewed at wileyonlinelibrary.com]

SCFAs, a bacterially produced metabolite, are associated with the pathophysiology and clinical outcomes of stroke (7). To determine whether SCFA levels were influenced by HT, the concentration of SCFA in biological samples was measured using high‐performance liquid chromatography‐tandem mass spectrometry (LC‐MS/ MS). SCFAs, including butyric acid (*p* = 0.004), propanoic acid (*p* = 0.011), iso‐butyric acid (*p* = 0.024), isovaleric acid (*p* = 0.047), valeric acid (*p* = 0.0003), and hexanoic acid (*p* = 0.017), decreased in the HG group compared to controls. The concentration of acetic acid has a decreased trend but no statistical significance in HG group than control rats (*p* = 0.061)(Figure 4A, H). In particular, valeric acid was significantly decreased in the fecal samples of HG rats compared to the NG group (Figure 4G). The level of butyrate acid has a decreased trend in HG group than in NG group but no statistical significance (*p* = 0.362, Figure 4D). Compared to the control group, the total SCFAs were decreased in HG group. Total levels of fecal SCFA were also reduced in the HG group compared to those in the NG group (Figure 4H).

### 3.4 Changes of gut microbiota and its metabolites are associated with inflammation and impaired BBB integrity in HT after stroke

**FIGURE 5:**
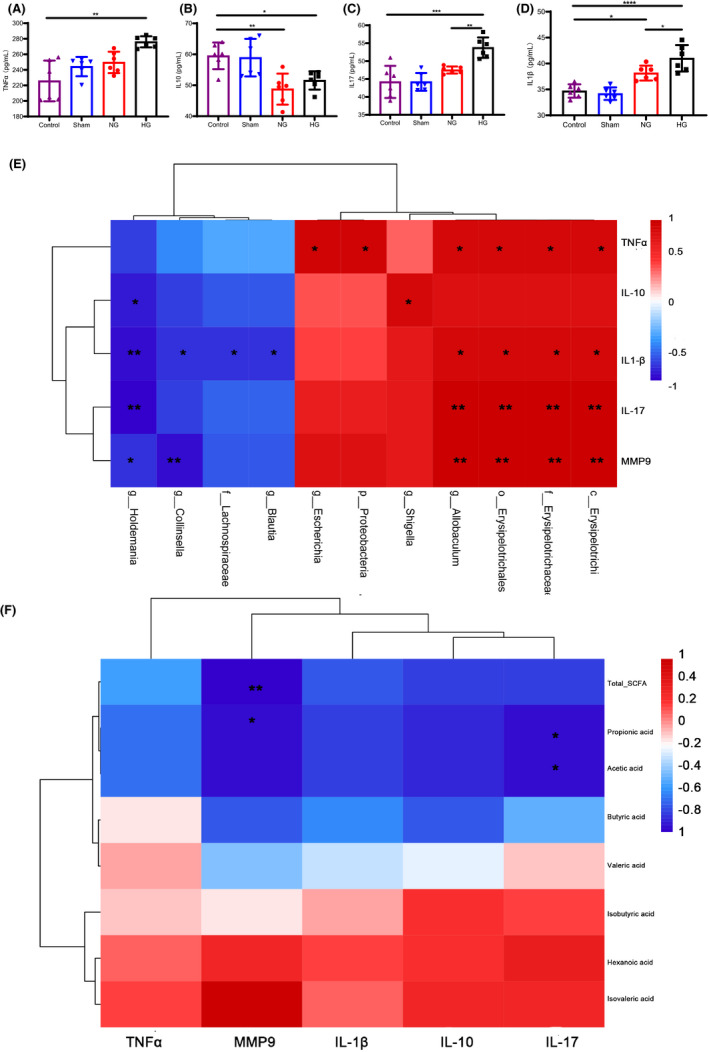
Correlation analysis of HT rats’ inflammatory factors, SCFAs, and microbiome. (A‐D) Detection of plasma inflammatory cytokine levels from diverse groups in rats. Plasma of rats from diverse groups were collected, respectively, for detection of IL‐1β, IL‐17, TNF‐α, and IL‐10 concentrations using ELISA kit (n = 6 per group). (E and F) Correlation between inflammatory cytokines, MMP‐9, and the level of SCFAs/key bacterial abundance performed by spearman correlation analysis (n = 6 per group). The color scale represents the strength of correlation, ranging from 1 (strong positive correlation) to −1 (strong negative correlation). Asterisks in each box indicate the corrected p‐value. Statistical analysis was performed by one‐way ANOVA analysis or Kruskal‐Wallis H test. Data are expressed as mean ± SD. ns: not significant, *p < 0.05, **p < 0.01, and ***p < 0.001 [Colour figure can be viewed at wileyonlinelibrary.com]

**FIGURE 6:**
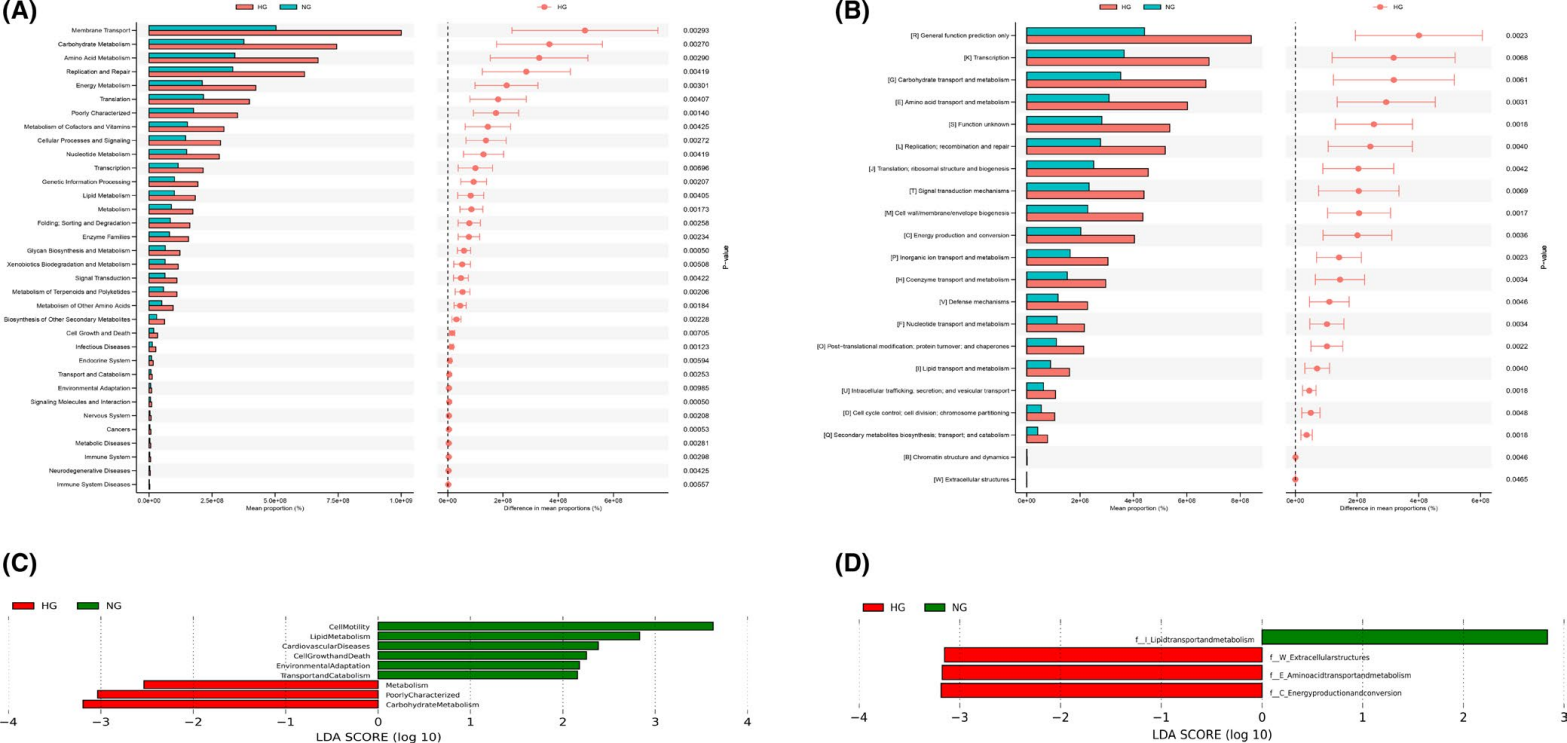
The functional prediction of gut microbiota based on KEGG and COG database in HG group. (A) The PICRUSt analysis based on KEGG database used to predict microbial metabolic function and analyze the functional differences. Mean proportion and their differences in predicted function of the gut microbiota between HG and NG group. (B) Study of functional group protein families based on COG databases between HG group and NG group. (C) LEfSe analysis was performed between the HG group and NG group to identify the differences of functional prediction based on KEGG database. Only the taxa meeting a significant LDA threshold value of >2.0 are shown. (D) Distinct COG pathways identified in the HG group and NG group using LEfSe analysis. Only the taxa meeting a significant LDA threshold value of >2.0 are shown [Colour figure can be viewed at wileyonlinelibrary.com]

The early BBB disruption modulated by MMPs in excessive neurovascular proteolysis is closely associated with HT events in ischemic stroke (29). BBB disruption was assessed by measuring MMP‐9 concentration. As shown in Figure 1C, higher MMP‐9 level was observed in the plasma of HT rats. In the NG group, lower MMP‐9 level was observed. Further analysis revealed a negative relationship between MMP‐9 and total SCFA (r = −0.943, *p* = 0.005) and propanoic acid concentrations (r = −0.886, *p* = 0.019) (Figure 5F). The butyric acid concentration has a trend of negative correlation with MMP‐9 level but no statistical significance (r = −0.714, *p* = 0.111; Figure 5F). In addition, the relative abundance of *Holdemania* and *Collinsella* was negatively correlated with MMP‐9 concentration (Figure 6E), while *Allobaculum*, Erysipelotrichi, Erysipelotrichales, and Erysipelotrichaceae were positively correlated with MMP‐9 levels (Figure 5E).

The inflammatory response is one of the key pathogenic events in HT after stroke (30). We analyzed the correlations between significant pro‐inflammatory and anti‐inflammatory cytokines and gut microbiota by Spearman's correlation (Figure 5E, F). As shown in Figure 5A–D, a significant increase in TNFα, IL‐17, and IL‐1β levels and decreased IL‐10 levels were observed in HG and NG rats compared with control, suggesting a pro‐inflammatory status under HT conditions. TNFα, IL‐1β, and IL‐17 concentrations were markedly higher in the HG group than in the NG group. However, plasma IL‐10 concentrations did not significantly differ between the HG and NG groups (Figure 5A–D). In the HG group, it was found that 11 differentially abundant taxons were negatively or positively associated with the parameters of HT, including inflammatory factors and MMP‐9. Thereinto, c_Erysipelotrichi, o_Erysipelotrichales, and f_Erysipelotrichaceae were positively associated with proinflammatory cytokines, including TNFα, IL‐1β, and IL‐17. *g_Holdemania*, *g_Collinsella*, f_Lachnospiraceae, and *g_Blautia* were on the contrary (Figure 5E). Some opportunistic and potentially pathogenic bacteria such as *g_Escherichia* and p_proteobacteria were positively correlated with the inflammatory cytokine TNFα levels (Figure 5E). Correlation analyses were next performed to evaluate the association between altered SCFAs and inflammatory response. As shown in Figure 5F, acetic acid and propionic acid were negatively associated with IL‐17 levels (r = −0.886, *p* = 0.019; r = −0.886, *p* = 0.019). Butyric acid was also negatively associated with the expression of TNFα, but there has no statistical significance (r = −0.143, *p* = 0.787).

### 3.5 Function characterization of the HT microbiome

We next performed the genus‐level functional profiling in HT using phylogenetic investigation of communities by Reconstruction of Unobserved States (PICRUSt). In particular, at level 2, 28 pathways were significantly different between the HG and NG groups (Figure 6A). LEfSe analysis showed the carbohydrate metabolism to be significantly more abundant in the HG group (Figure 6C). In addition, we identified 19 COG metabolic pathways differentially abundant at level 2 between the HG and NG groups (Figure 6B). LEfSe analysis also identified energy production and conversion to be significantly more abundant in the HG group than in the NG group (Figure 6D).

### 3.6 HT induced by hyperglycemia is microbiota dependent

**FIGURE 7:**
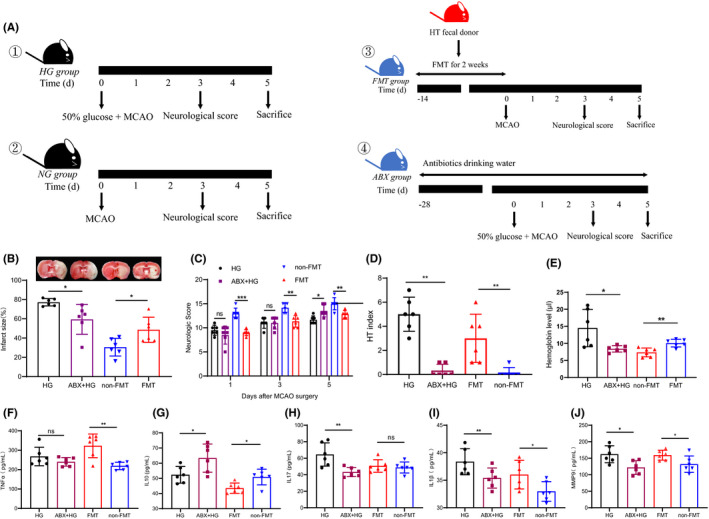
Hyperglycemia‐induced HT pathogenesis is dependent on gut microbiota and HT susceptibility of MCAO rats is transmissible. (A) Experimental design. FMT: fecal microbiota Transplantation; ABX: antibiotics; HG: hyperglycemia group, NG: normoglycemia group. (B) Rats were euthanized on day 5, and infarct size was measured. The macroscopic HT was evaluated by representative images of TTC staining of the brain slices of different groups (n = 6 per group). (C) Neurologic score changes were monitored on 1d, 3d, and 5d after MCAO surgery. (n = 6 per group). (D) Macroscopic HT assessed using HT index in different groups (n = 6 per group). (E) The hemoglobin content in the ischemic hemisphere which is used as a measure of the severity of bleeding (n = 6 per group). (F to I) The plasma expression of proinflammatory cytokines IL1β, IL‐17, TNFα, and IL‐10 was measured by ELISA kit (n = 6 per group). (J) MMP‐9 level in different groups (n = 6 per group). Data represent means ± ‐SD; *p < 0.05, **p < 0.01, and ***p < 0.001 by unpaired Student's t test or Mann‐Whitney rank sum test [Colour figure can be viewed at wileyonlinelibrary.com]

Since targeted microbiome modification by an ABX cocktail had a neuroprotective effect after stroke (24), we hypothesized a similar effect on HT. To determine whether the altered microbiota was responsible for heightened HT pathogenesis, we treated hyperglycemia‐injected rats with broad‐spectrum ABX cocktail for 4 weeks. As a control, one hyperglycemia‐injected group of rats was not treated with ABX. ABX‐treated and untreated rats were subjected to HT (Figure 7A). ABX treatment effectively depleted the microbial load in the gut (24). Compared with untreated controls, ABX reduced brain infarction, macroscopic HT, hemoglobin level, and neurological scores (Figure 7B–E). The MMP‐9 level (*p* = 0.014) and the expression of pro‐inflammatory cytokines, including IL1β (*p* = 0.036) and IL‐17 (*p* = 0.006), were similarly reduced in ABX‐treated rats (Figure 7F–J). In addition, IL‐10 expression was higher in the ABX + HG group than in the HG group (*p* = 0.031).

### 3.7 Hyperglycemia‐induced HT susceptibility is transmissible

Because HT promotes the growth of proinflammatory bacteria, we investigated whether the altered gut microbiota in HT rats was sufficient to induce worsened HT. To address this concern, we induced HT in rats via acute hyperglycemia. After 5 days, feces from HT rats were homogenized and orally inoculated into rats with ABX‐related gut microbiota depletion. Two weeks after fecal transplantation, the recipient rats underwent MCAO (Figure 7A). Although high glucose was not injected intraperitoneally before MCAO procedure, rats that received microbiota from HT donor rats developed higher brain infarction, higher HT index, increased hemoglobin level in ischemic hemisphere, macroscopic petechiae, and exhibited markedly severe neurological scores as compared to control rats (MCAO rats without fecal transplantation) (Figure 7B–E). Consistent with clinical features, rats receiving fecal microbiota from HT donors showed increased TNFα levels (*p* = 0.002) and IL‐1β levels (*p* = 0.039) and decreased IL‐10 concentrations (*p* = 0.016), indicating markedly severe inflammatory responses, accompanied by higher MMP‐9 levels (*p* = 0.044) (Figure 7F–J).

## 4 DISCUSSION

**FIGURE 8:**
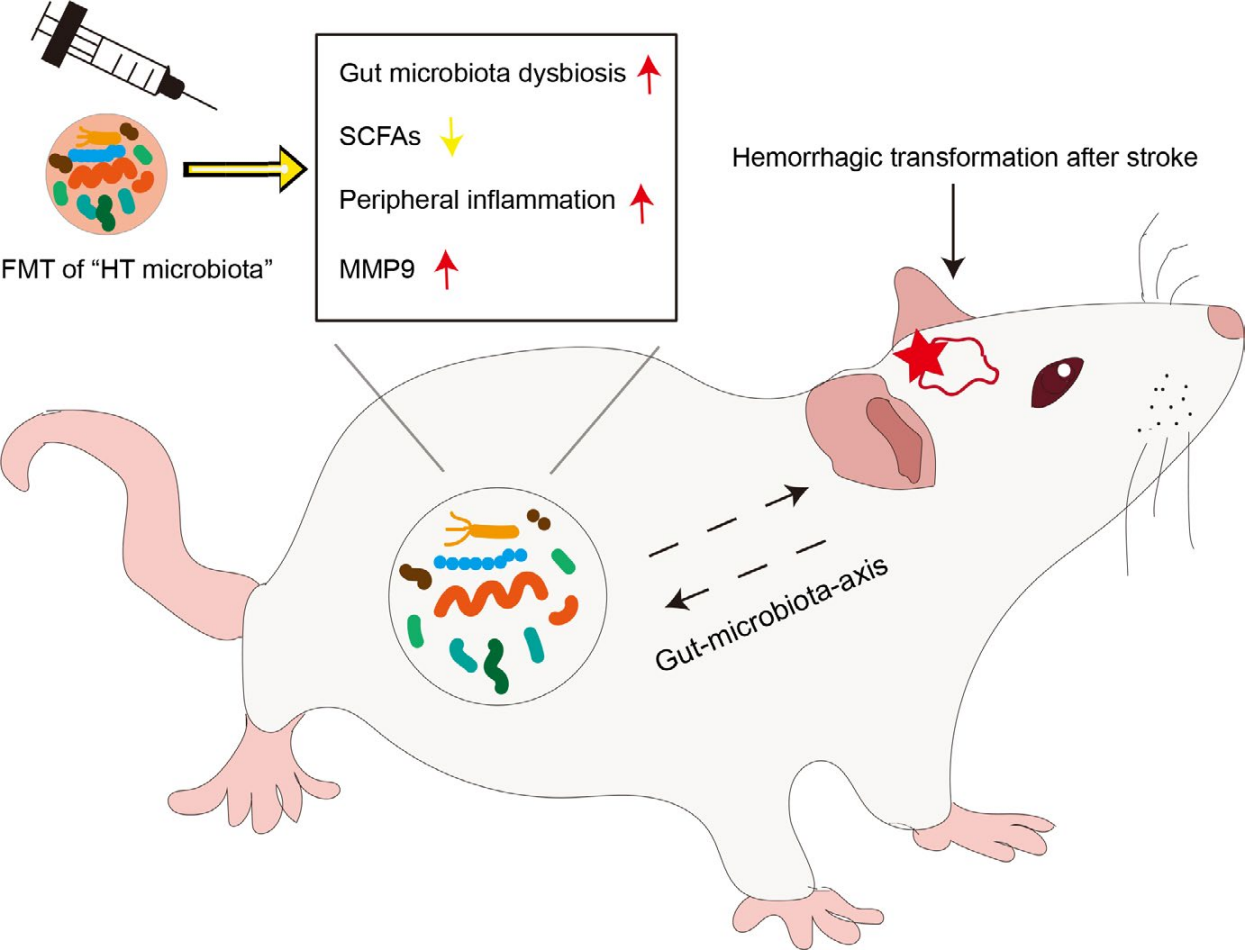
The gut microbiota modulates the inflammatory response and MMP‐9 that are associated with hemorrhagic transformation after stroke. Hemorrhagic transformation (HT) leads to gut dysbiosis and the inflammatory response. The changes of gut microbiota, peripheral inflammation, and SCFAs may further regulate MMP‐9 level and affect blood‐brain barrier permeability, contributing to an increase of HT susceptibility after stroke. MCAO rats that received fecal microbiota transplantation (FMT) of the “HT microbiota” exhibited high risk of HT [Colour figure can be viewed at wileyonlinelibrary.com]

Our study addressed the ongoing question of the relationship between gut microbiota and stroke. We observed that the changes in the microbiota composition were associated with the severity and risk of HT after stroke (Figure 8). Sequencing of the gut microbiota showed an increase in the relative abundance of Proteobacteria and Actinobacteria in the HG group compared to the NG group. In addition, compared to NG group, HG group enriched pathogenic and opportunistic pathogens such as Erysipelotrichi, *Escherichia*, Streptomycetaceae, and Pseudonocardiaceae. In particular, specific changes in gut microbiota and the concentrations of certain organic acids were linked to immunological alterations and MMP‐9.

Although hyperglycemia is a risk factor for HT after stroke, its precise mechanism is poorly understood. This study suggests an important mechanism that might explain, if confirmed in humans, the association of acute hyperglycemia with increased HT incidence. Oxidative stress plays a vital role in HT pathology. Preischemic hyperglycemia aggravates brain damage by enhancing the formation of reactive oxygen species (ROS) during the early reperfusion period (31), while stress before MCAO could induce anaerobic gram positive bacterial species such as *Enterococcus faecalis*, *Staphylococcus aureus*, and *Propionibacterium aureus* (32). Fecal Enterobacteriaceae, as a large family of Gram‐negative bacteria, is higher in a murine model of type 2 diabetes (*db*/*db*) than in phenotypically normal littermates (*db*/+) after stroke. Microbiota‐derived lipopolysaccharide (LPS), associated with Enterobacteriaceae, is translocated into circulatory system and induces the release of inflammatory cytokines by interacting with toll‐like receptor 4 (TLR4) (33). In turn, the overgrowth of Enterobacteriaceae accelerates systemic inflammation and nitrate respiration through the LPS‐TLR4 pathway to exacerbate brain infarction (34). In the present study, at the phylum level, HT significantly increased the abundance of anaerobic bacteria such as Actinobacteria, Proteobacteria, Verrrucomicrobia, and synergistetes. In addition, HT rats showed enrichment of fecal Proteobacteria (this phylum contains Enterobacteriaceae). Some scholars have proposed that hyperglycemia increases the formation of free radicals and reduces oxygen production (31). On the other hand, oxidative stress also reduced the oxygen content in the intestinal tract and caused perturbed cecal microbiota with increased levels of *Clostridiales*, *Lactobacillus*, *Treponema*, and *Oscillospira* in mice (35). Thus, HT induced by hyperglycemia may inhibit the oxygen formation in the intestinal tract and increase the production of anaerobic bacteria. Nevertheless, future studies are required to investigate the number of reactive oxygen and nitrogen species (RONS) and the underlying interaction of gut microbiota and oxidative stress.

Accumulating evidence has shown that the gut microbiota is a pivotal risk factor for stroke by influencing immune homeostasis (36, 37). Acute brain ischemia contributes to gut microbiota dysbiosis, which in turn affects stroke outcome via a local neuroinflammatory reaction and peripheral homeostasis (38). Dysbiosis is causally linked to deteriorated stroke outcome. Recolonizing germ‐free mice with poststroke microbiota exacerbates lesion volume and increases the expression of proinflammatory IL‐17 and IFN‐γ cytokines (38). In our study, we have also observed a substantial induction of proinflammatory cytokines by transfer of a dysbiotic microbiome. The aggravation of immune and inflammatory responses in HT rats may be explained by our microbiota analysis showing that the F/B ratio and abundance of Proteobacteria and Actinobacteria, these two pro‐inflammatory phylum highly abundant in stroke patients (39) and other neurological immune diseases (27), is increased under hyperglycemia‐induced HT. Recent studies have documented detrimental roles of *Parabacteroides* in human health (40). However, there is also substantial evidence that *Parabacteroides* promotes stroke pathogenesis. Colonization of *Parabacteroides* into GF mice led to the severe brain injury and the induction of inflammatory cytokines (41). In addition to *Parabacteroides*, several other pathogenic bacteria or conditional pathogens, including *Butyricimonas*, *Desulfovibrio*, *Fusobacterium*, *Erysipelotrichaeae*, *papillibacter*, and *Veillonellaceae*, promote inflammatory responses after cerebral infarction (42). Our microbiota analyses demonstrated that some of these inflammatory bacteria, such as *Erysipelotrichaceae*, *Butyricimonas*, and *Desulfovibrio*, were highly abundant in the HT rats gut. Higher abundance of pro‐inflammatory bacteria in the HT rats is clinically relevant because patients with stroke and the higher risk factors of stroke exhibit higher level of pro‐inflammatory cytokines.

Bidirectional signaling consisting of top‐down communication and bottom‐up signaling occurs between the gut and the brain in stroke, the so called “gut‐brain axis” (43). The brain‐gut barriers might be new perspective mechanisms of stroke. The BBB plays the vital role in protection against proinflammatory response and the important pathophysiological changes to HT. Herein, we demonstrate that hyperglycemia increased the level of MMP‐9, levels related to the disrupted BBB integrity in MCAO rats, which may allow opportunistic pathogens or the toxic products of gut microbiota to come in close proximity to the brain. MMP‐9 is known to play a key role in cerebral ischemia reperfusion and may be a new therapeutic target (15). In addition to that gut microbiota and its metabolites affect BBB permeability, several bacterial species including *Helicobacter pylori*, *Prevotella sp*, *Clostridium sp*., *Streptococcus sp*., *Bacteroides sp*., *Bifidobacterium*, and *Akkermansia* belongs to mucin‐degrading bacterium and produce mucolytic enzymes (44). Intestinal permeability was disturbed due to cerebral ischemic stroke and associated with the severity of stroke (42). Chen et al. demonstrated that the brain and gut barriers were highly correlated (42). Our microbiota analyses demonstrated that mucolytic bacteria such as *Prevotella*, *Akkermansia*, *Streptococcus*, and *Helicobacter* are highly abundant in the HG group. We should investigate the intestinal permeability in HG group to verify the role of these mucolytic bacteria. Whether the gut microbiota influences MMP‐9 levels and BBB requires further research.

The gut microbiota is known to influence host homeostasis, in part via metabolic pathways such as SCFAs, amino acid metabolism, and bile acid metabolism (28). In the present study, we found changes in the gene functions of bacteria from hyperglycemic rats, especially in physiological metabolic functions including carbohydrate, amino acid, and fatty acid metabolism. The *Bacteroides* and *prevotella* have been associated with diets enriched in plant fibers and animal carbohydrates, respectively (45). The lack of these types in HT rats might decrease the capacity of gut microbe‐mediated metabolism. Previous studies have shown that two‐day exposure to a high‐sugar diet could rapidly alter gut microbial composition and depletes SCFAs (21). Transplantation of SCFA‐producing bacteria into GF mice or antibiotic‐depleted mice was shown to improved outcomes after stroke, probably because SCFAs, particularly butyrate, improve neurogenesis (46) and attenuate brain inflammation (47). In this study, an especially meaningful change included the loss of SCFA producing microbes belonging to the Ruminococcaceae and Lachnospiraceae families in the HG group compared to the NG group. This family is capable of producing various SCFAs, including acetate and butyrate. Synergistetes, which can utilize acetate and amino acids (48), as well as be associated with soft‐tissue infections (49), was increased in the HG group. Concomitant with the significant change in gut microbial composition, the level of total SCFAs, especially butyrate, was decreased in HG group. Butyrate is essential for modulating the immune system and maintaining the integrity of intestinal epithelium. In the present study, after HT, SCFA levels correlated with inflammatory cytokines including TNF‐α, IL‐1β, and IL‐17. The genus of *Holdemania* was increased in HG group, which is correlated with anxiety, neuroinflammation (50), and impaired lipid and glucose metabolism (51). Depletion in butyrate‐producing taxa has been linked to the high risker of stroke including type 2 diabetes (T2D), obesity, and cardiovascular disease (52). Whether the addition of SCFAs including acetate, butyrate, or the mixture has a reversible effect on microbial community composition in either the stool or cecum of HT rats, followed by an attenuating effect on the inflammation status of the host after stroke, even HT, requires after research.

Post‐stroke cognitive impairment is known as one of common complications of stroke. It often coexists in stroke patients with adverse effects on patient outcome (53). Various neurological disorders including stroke and Alzheimer's disease involve the changed gut‐brain axis and similar cognitive decline (54). Compared to the group with greater Bacteroides abundance, the Prevotella group showed more negative emotional responses and reduced functional activation of the hippocampus (55). An antibiotics cocktail treatment abolished the alleviation of cognitive impairment afforded by intermittent fasting, demonstrating the crucial role of gut microbiota in cognitive deficits (56). Recently, Liu et al have demonstrated the relationships among gut microbiota and neuropsychological characteristics in amnestic mild cognitive impairment patients (57). The producing‐SCFAs bacteria including class Clostridia, phylum Firmicutes, including the families Lachnospiraceae, genus *Blautia* and *Ruminococcus* were decreased in amnestic mild cognitive impairment patients (57). In the present study, the relative abundance of Lachnospiraceae, Clostridiaceae, and *Ruminococcus* is less in HG group than in NG group. It is vital to further identify the role of brain‐gut‐ microbiota interactions in cognitive impairment after hemorrhagic transformation.

Human microbiota plays an essential role in cerebrovascular diseases. Recent advances in DNA sequencing, proteomics, metabolomics, and computational tools are dramatically increasing access to the identification of host‐microbiota interactions in cerebrovascular diseases (58). Although the actual link between microbiota and the underlying mechanisms are yet to be characterized, the manipulation of gut microbiota appears as a promising future‐therapy in stroke and its complications (43). In antibiotic‐treated MCAO animals, gut microbiota exerts protective effects on survival, which may be associated with systemic immunodepression (33, 43, 58). In our study, we have also demonstrated the protective and anti‐inflammatory effects of antibiotic treatment on HT after stroke. Based on the association of gut microbiota and stroke shown in previous researches and our results, there are many kinds of possible therapeutic avenues such as antibiotic eradication, microbial‐derived metabolites, and immune modulation.

This study has a few limitations. First, it involved animal model. The composition of the gut microbiota in rodents is not with the same as that of humans. The limits of research in this topic, the “man versus mouse” question, cannot be underestimated (43). Therefore, the observed effects of HT on human gut microbiota composition and SCFA levels need to be validated in clinical settings. Furthermore, the use of PICRUSt instead of full metagenomic shotgun sequencing limited for assessing the functional aspects of the gut microbiota. In addition, the precise function of some affected bacterial species in rats with hyperglycemia‐induced HT is unknown and should be investigated in future studies.

In summary, we found that HT after stroke changes microbial composition and organic acid concentrations in the gut; changes in turn associated with metabolic and inflammatory biomarkers. The current findings contribute to our understanding of the critical cause of the increasing incidence of HT under hyperglycemia during the acute stage of the onset of cerebral ischemia. However, further studies are needed to determine whether these alterations might serve as predictive indicators of HT after ischemic stroke.

## CONFLICT OF INTERESTS

All authors confirm that they have no conflicts of interest.

## AUTHORS’ CONTRIBUTIONS

JX and QH designed the experiments, drafted the work, performed the overall data analysis, and wrote the manuscript; QH and DL contributed to the animal experiments; QH and MPW contributed to 16S sequencing of gut microbiota, data analysis, and figure generation; FY and ZYL contributed to literature search, data collection, analysis, and interpretation; XJF and YFL provided technical support and assisted with data collection and data interpretation; JX critically revised the paper. All authors approved the final version of the paper.

## Supporting information

Figure S1

Figure Legend

## Data Availability

The datasets generation for this study can be found in the NCBI (https://www.ncbi.nlm.nih.gov/; BioProject: PRJNA 736329).
