# Supplementary figures and images for: Alterations in the gut microbiome with hemorrhagic transformation in experimental stroke
Source: CNS Neurosci Ther. 2021 Sep 30;28(1):77–91. doi: 10.1111/cns.13736 (PMC8673707; doi:10.1111/cns.13736)

 Control     HG

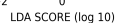

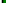 Control

 HG

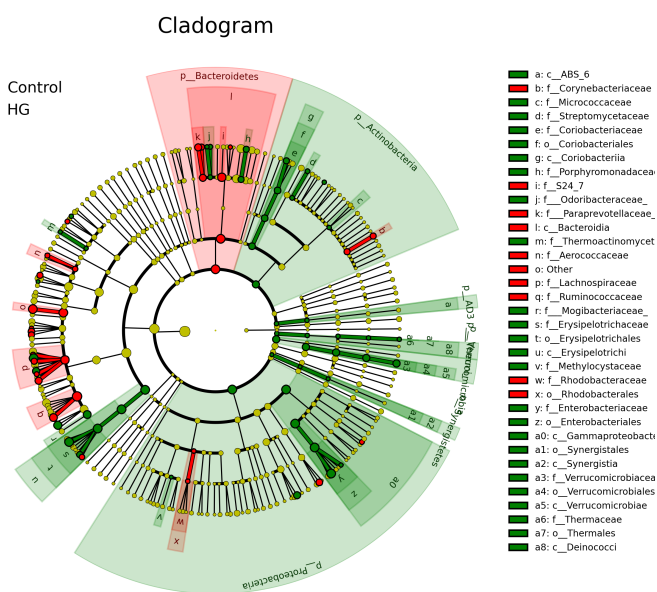

Supplement: Supplementary file 1 — Figure S1 [file CNS-28-77-s002.pdf]
