# Supplementary material for: Alterations in the gut microbiome with hemorrhagic transformation in experimental stroke
Source: CNS Neurosci Ther. 2021 Sep 30;28(1):77–91. doi: 10.1111/cns.13736 (PMC8673707; doi:10.1111/cns.13736)
Supplement: Supplementary file 2 — Figure Legend [file CNS-28-77-s001.docx]

**Significantly discriminative taxa between HG and the control groups determined using linear discriminant analysis effect size (LDA effect size)**. (A) Taxa were sorted by degree of difference. Only the taxa meeting a significant LDA threshold value of >2.0 are shown. (B) the overall representation of bacteria composition in HG and NG groups by cladogram. All species with a relative abundance of less than 1% and classified as ‘‘unclassified’’ and ‘‘unidentified’’ were classified as ‘‘Others’’ (n=6 per group).
